# Modeling Self-Healing of Concrete Using Hybrid Genetic Algorithm–Artificial Neural Network

**DOI:** 10.3390/ma10020135

**Published:** 2017-02-07

**Authors:** Ahmed Ramadan Suleiman, Moncef L. Nehdi

**Affiliations:** Department of Civil and Environmental Engineering, Western University, London, ON N6A 5B9, Canada; asuleim3@uwo.ca

**Keywords:** autogenous, self-healing, crack width, artificial neural network, genetic algorithm

## Abstract

This paper presents an approach to predicting the intrinsic self-healing in concrete using a hybrid genetic algorithm–artificial neural network (GA–ANN). A genetic algorithm was implemented in the network as a stochastic optimizing tool for the initial optimal weights and biases. This approach can assist the network in achieving a global optimum and avoid the possibility of the network getting trapped at local optima. The proposed model was trained and validated using an especially built database using various experimental studies retrieved from the open literature. The model inputs include the cement content, water-to-cement ratio (w/c), type and dosage of supplementary cementitious materials, bio-healing materials, and both expansive and crystalline additives. Self-healing indicated by means of crack width is the model output. The results showed that the proposed GA–ANN model is capable of capturing the complex effects of various self-healing agents (e.g., biochemical material, silica-based additive, expansive and crystalline components) on the self-healing performance in cement-based materials.

## 1. Introduction

Concrete is considered the world’s most widely used construction material. Although it is cost-effective and able to carry relatively high compressive loads, concrete is susceptible to micro-cracks, which can jeopardize the durability of civil infrastructure, inflicting multibillion dollar losses in premature degradation. Several factors can lead to crack formation in a concrete matrix, including mechanical load, restrained shrinkage or thermal deformation, differential settlement, poor construction methods and faulty workmanship. Therefore, harmful substances such as chloride ions, sulfates, and carbon dioxide can easily ingress into to the concrete matrix, leading to reinforcing steel corrosion and concrete damage. Moreover, conventional concrete repairing and rehabilitation techniques are time consuming and often not effective. According to Li and Herbert [1], the cost of repair and rehabilitation of existing civil infrastructure, especially in developed countries, has exceeded the cost of building new infrastructure. For instance, a recent report published by the American Society of Civil Engineers (ASCE), awarded the current status of the American national infrastructure a grade of D+ (equivalent to poor condition). It also estimated that $US 3.6 trillion is needed to repair the aging infrastructure by the year 2020. Indeed, the deteriorated civil infrastructure not only drains financial resources, but also has social and environmental implications.

In recent years, research on the ability of concrete to heal itself has received increasing attention. The inspiration came from the concept of biomimicry and the healing process in living nature [2,3]. For instance, when the skin of humans or animals is injured due to cuts, scrapes, or scratches, it can repair itself biologically. Although it is almost impossible to artificially simulate this exact biological healing process in concrete, several studies have proved that a Portland cement concrete matrix can heal itself intrinsically [4,5,6,7,8,9,10,11,12].

Different mechanisms are responsible for autogenous (or intrinsic) self-healing in concrete, including (a) further hydration of anhydrous cement or cementitious minerals; (b) carbonation of calcium hydroxide; (c) expansion of the hydrated cement due to the swelling of calcium silicate hydrate; and (d) precipitation of impurities from the ingress water and spalling of loose concrete particles in cracks [13,14,15,16]. Nevertheless, both continuous hydration and carbonation of calcium hydroxide are considered as the major mechanisms of autogenous self-healing in concrete [17].

Autogenous self-healing in concrete can also be stimulated by incorporating different healing agents into the concrete matrix. For instance, previous studies by Termkhajornkit et al. [18], Şahmaran et al. [19] and Van Tittelboom et al. [17] showed that partially replacing Portland cement with supplementary cementitious materials, such as blast-furnace slag and fly ash, can improve the self-healing phenomenon in the cementitious matrix. In addition, a study by Sisomphon et al. [11] reported that using expansive and crystalline additives could also enhance self-healing.

Several other studies investigated the possibility of incorporating bio-chemical self-healing agents in concrete to promote the self-healing efficiency [20,21,22,23,24,25,26,27,28]. For instance, Jonkers et al. [21] investigated the potential of using a certain type of alkali-resistant spore-forming bacteria as a self-healing agent. Spore-forming bacteria related to the genus Bacillus were added into the cementitious material. It was shown that the use of bacteria in concrete can help fill micro-cracks. In addition, a study conducted by Van Tittelboom et al. [24] reported that the use of bacteria can help reduce water permeability into concrete. Wiktor and Jonkers [27] studied the effect of combining bacteria spores and calcium lactate in concrete. They found that the combined effect can significantly enhance the concrete’s ability to self-heal its cracks independently.

Therefore, the development of self-healing in concrete depends on numerous factors and parameters which are highly interdependent and exhibit substantial complexity, having combined roles in the self-healing efficiency of concrete. This makes it difficult to model and predict the self-healing effect of concrete given such complex multitude of parameters.

One promising approach to predicting the self-healing efficiency of concrete is artificial intelligence techniques, such as artificial neural networks (ANNs). According to Adeli [29], ANNs offer a reliable tool that can model and predict complex problems. It basically consists of computational devices inspired by biological learning in the brain. It has a self-learning capability able to capture complex interactions between different variables. According to Kartam et al. [30], ANNs can be applied to a variety of tasks and problems, such as classification, interpretation, diagnosis, modeling, and control. They are more suitable to problems that are highly complex to solve by mathematical modeling or other classical procedures [29].

Since 1989 when the first article on the application of ANNs in civil engineering was published, several studies have reported on the excellent ability of ANNs to model and solve complex problems in different civil engineering areas [31,29]. For instance, ANNs have been successfully applied to investigate the concrete’s compressive and shear strength, strain, dynamic modulus of elasticity, chloride permeability, crack pattern, and autogenous and drying shrinkage [32,33,34,35,36,37,38,39,40,41,42,43]. In most cases, the ANN model was trained using the back-propagation (BP) algorithm. Basically, BP is a local search algorithm used in combination with gradient descent to update the weights and biases of the neural network and minimize the performance function [44]. According to Huang et al. [45], training an ANN by BP has been a successful approach that can provide solutions for several engineering applications. However, due to the random initialization of weights and biases, the BP algorithm could be trapped in local optima and may not find the global optimum [44,45].

Recently, a number of studies combined ANN modeling with genetic algorithms (GA) to improve the convergence to global optimum [46,47,48]. Therefore, in the present study, the feasibility of using a hybrid genetic algorithm–artificial neural network (GA–ANN) for predicting the autogenous self-healing in concrete is investigated.

## 2. Research Significance

Concrete structures are generally vulnerable to cracking, which can adversely affect its performance and shorten its service life by primarily allowing harmful substances such as chloride ions, sulfates, and carbon dioxide to ingress into to the cementitious matrix [49,50,51]. In addition, periodic maintenance and repair can be very costly, especially for large-scale structures. Thus, designing concrete structures with self-healing ability could save billions of dollars in maintenance and repair costs and improve both concrete durability and sustainability, thus leading to eco-friendly civil infrastructure. In this paper, an attempt was made to develop a hybrid artificial intelligence-based model to accurately predict the concrete’s capability to heal its own cracks. A comprehensive database of concrete crack healing was created and used to train the proposed GA–ANN model. The model thus developed was able to predict the ability of concrete to self-heal its own cracks with adequate accuracy. This could allow tailoring self-healing strategies for enhancing the durability design of concrete, thus leading to reduced maintenance and repair costs of concrete civil infrastructure.

## 3. Concept of Neural Network Prediction of Self-Healing in Concrete

Several strategies of self-healing in concrete have been introduced. According to Van Tittelboom and De Belie [52], such strategies can be classified based on the corresponding healing mechanisms into three groups: intrinsic healing, capsule-based healing and vascular healing. Intrinsic healing includes autogenous healing (further hydration of un-hydrated cement and carbonation of calcium hydroxide) and improved autogenous healing via agents or approaches that can promote further crystallization and cementitious hydration reactions. Strategies such as capsule-based healing (e.g., microcapsules filled with a healing agent such as epoxy and added during concrete mixing) or vascular healing (networks of hollow tubes built into the cementitious matrix) do not always require interaction with concrete components to promote self-healing. Since each of these self-healing strategies has a different approach and deals with a different mechanism, it is reasonable to model each separately. Thus, in this paper, intrinsic self-healing alone will be considered to develop a GA–ANN model capable of predicting the crack-self healing in cementitious materials. Studies retrieved from the literature were carefully selected based on reporting measurements of the change in crack-width due to self-healing under similar environmental conditions (i.e., water submersion). For instance, Özbay et al. [53], Sahmaran et al. [54], and Van Tittelboom et al. [17] investigated crack self-healing under different environmental conditions; however, to obtain reliable model predictions, only results of self-healing obtained for completely water-submerged specimens were retained for developing the GA–ANN model. Sisomphon et al. [11] and Wiktor and Jonkers [27] also investigated the self-healing potential of cement-based materials under similar environmental conditions and their data were thus used. In addition, different agents were used to promote self-healing. For instance, Özbay et al. [53], Sahmaran et al. [54], and Van Tittelboom et al. [17] investigated the autogenous self-healing of concrete incorporating supplementary cementitious materials. Wiktor and Jonkers [27] improved the autogenous healing by incorporating bio-chemical self-healing agents [52]. Two biochemical agents consisting of a mixture of bacterial spores and calcium lactate were used. Chahal et al. [20] studied the effect of bacteria on the compressive strength, water absorption and rapid chloride permeability of fly ash concrete. They found that the properties of concrete made with fly ash along with an optimized dose of bacteria could be improved. Sisomphon et al. [11] also studied the self-healing of cement-based materials incorporating calcium sulfo-aluminate-based expansive additive and a crystalline additive. Although in these studies different healing agents were used, the main healing product formed in the cracks were calcium carbonate (CaCO_3_) and/or calcium silicate hydrate (C–S–H). For example, both Özbay et al. [53] and Sahmaran et al. [54] used scanning electron microscopy (SEM) and X-ray diffraction (XRD) to investigate the mineralogy and chemical composition of the healing product observed in the cracked specimens exposed to continuous curing. They reported the formation of both CaCO_3_ and C–S–H. In addition, a high amount of CaCO_3_ was reported in the case of using supplementary materials with higher CaO content. Wiktor and Jonkers [27] also reported significant formation of CaCO_3_ as a healing product due to metabolic conversion of calcium lactate and the reaction of metabolically produced CO_2_ molecules with Ca(OH)_2_ minerals present in the concrete. Sisomphon et al. [11] also found that CaCO_3_ was the major healing product formed in cracks due to the increased release of Ca^2+^ and high pH of the cement mortar specimens incorporating the healing agents. According to Van Tittelboom and De Belie [52], intrinsic healing can include autogenous healing (further hydration of un-hydrated cement and carbonation of calcium hydroxide) and improved autogenous healing using agents or approaches that promote crystallization and more cementitious hydration reactions. Özbay et al. [53], Sahmaran et al. [54], and Van Tittelboom et al. [17] investigated autogenous self-healing under similar environmental condition and reported practically similar final healing products. Moreover, studies by Sisomphon et al. [11] and Wiktor and Jonkers [27] investigated the improved autogenous self-healing under similar condition and reported similar final healing products. Hence, developing a model based on artificial neural networks to predict the effect of such agents reported in these studies on crack self-healing was considered a suitable approach.

## 4. Artificial Neural Network (ANN)

### 4.1. Neural Network Approach

**Figure 1:**
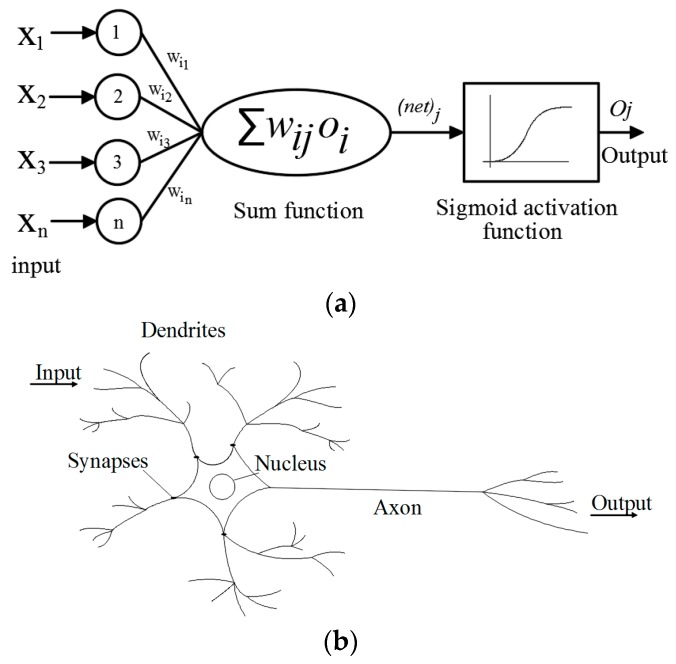
Schematic illustration of (**a**) artificial neuron; and (**b**) biological neuron.

Figure 1 displays a schematic illustration of both artificial and biological neurons. An artificial neural network is a highly interconnected network of parallel distributed possessors or neurons that has a learning process similar to the extent of the learning procedure in a biological brain [29,30,40,55,56,57,58,59]. It operates as a black box through a learning process with the ability to synthesize and memorize complex data structure [29].

ANN modeling has been applied in almost all engineering fields. For civil engineering in particular, ANN modeling has been employed to solve complex problems in the areas of structural, construction, geotechnical, environmental, and management engineering [29,30]. For instance, Jiang et al. [60] modeled the microbial-induced corrosion of concrete sewers using the ANN approach. This type of corrosion involves complex mechanisms that are difficult to model analytically. The ANN model provided accurate estimations in comparison to multiple regression models. Similarly, Venkiteela et al. [41] developed an ANN model capable of predicting the dynamic modulus of elasticity of concrete at an early age with reasonable accuracy. Since self-healing in concrete is a multifaceted process that requires a powerful modeling tool, ANN was considered in the present study in an attempt to capture the interdependent parameters influencing the self-healing mechanism and its high level of complexity.

### 4.2. Neural Network Architectures and Parameters

**Figure 2:**
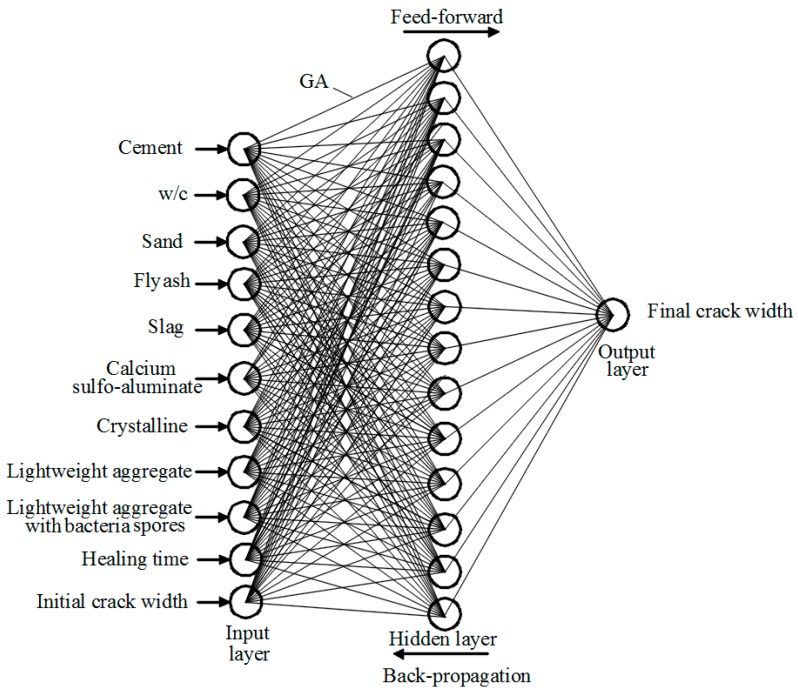
Architecture of genetic algorithm–artificial neural network (GA–ANN) model.

Since there is no commonly accepted optimal method to determine the best architecture of an ANN, a trial and error approach was adopted. The design of the network architecture starts with fewer hidden neurons, and then the number of hidden neurons is adjusted. The network architecture that provided best generalization was retained and is illustrated in Figure 2. It consists of 11 input neurons representing the main parameters influencing the self-healing of concrete, along with one hidden layer comprising 14 neurons, and one output layer with a single neuron representing the crack width as an indication of self-healing.

There are several different classes of architectures in neural networks in which neurons (nodes) are structured. For instance, in a feed-forward network, nodes are structured in parallel multilayers that can be classified into input layer, hidden layers, and output layer [39,40].

Each node mainly consists of three elements where the input information passes through, including connecting links or weights, summing-junction, and activation function [39,55,61]. Weights in a neural network basically represent the strength between the connected nodes. For instance, when a node in the input layer receives information from an external environment, the output will send it as an input to the neighboring node at the next layer (i.e., hidden layer) multiplied by the weight value. Thereafter, the summing-junction combines all the weighted products. Equation 1 refers to the weighted sums of the input components [55,61,62].

(1) $$l_{n}=\overset{p}{\underset{j=1}{\sum }}W_{nj}x_{j}+b$$

where *l_n_* is the weighted sums of the input component, *W_nj_* is the weight between neurons, *x_j_* is the input, and *b* is the bias.

The summation process will form a single input, which will be adjusted by an activation function [40,61,62]. Basically, the activation function simulates the firing rate of the neuron to axon in the biological brain. Thus, in the computational model, the final single output of a neuron can be calculated based on the following equation:

(2) $$y_{n}=A_{f}\left(l_{n}\right)$$

where *y_n_* is the output of the neuron and *A_f_* is the activation function.

There are several common activation functions that allow neural networks to solve difficult problems, including the sigmoid, ramp, and Gaussian functions. In the case of using a multilayer neural network with receptive fields, using the sigmoid function as an activation function is generally recommended (e.g., [61]). In the current paper, the tansigmoid function shown in Equation (3) was used in the hidden layer and a pure linear transfer function as shown in Equation (4) was used in the output layer neurons.

(3) $$\mathrm{tansig}\left(x\right)=\frac{2}{1+e^{-2x}}$$

(4) purelin(x)=x

### 4.3. Hybrid Genetic Algorithm–Artificial Neural Network (GA–ANN)

Table 1 shows the values of parameters used in the GA–ANN model. Genetic algorithms are powerful optimization tools based on Darwin’s natural selection and evolution theory. It has the capability to find the global optima through stochastic search techniques in a large solution domain. The process of the GA consists of steps including evaluation, selection, crossover, and mutation. Therefore, implementing a GA in ANN can improve the prediction accuracy of the ANN model. Several studies have reported that using GA–ANN can provide a reliable solution for different engineering optimization problems. For instance, Zhang et al. [63] showed that using artificial neural network−genetic algorithm-based optimization provided higher accuracy prediction for the effect of pH, carbodiimide concentration, and coupling time on the activity yield of immobilized cellulose on the smart polymer. Yasin et al. [64] used a hybrid artificial neural network–genetic algorithm approach to optimize the removal of lead ions from aqueous solutions using intercalated Tartrate-Mg–Al layered double hydroxides. It was shown that a small residual error existed between the predicted and experimental values. Ho and Chang [65] investigated the feasibility of using an artificial neural network model with a genetic algorithm to predict the platelet transfusion requirements for acute myeloblastic leukemia patients. A genetic algorithm was applied in the ANN model to optimize the weights and biases governing the input-output relationship of the ANN model. They found that using a hybrid (GA–ANN) model effectively predicted the transfusion requirement of the acute myeloblastic leukemia patients.

**Figure 3:**
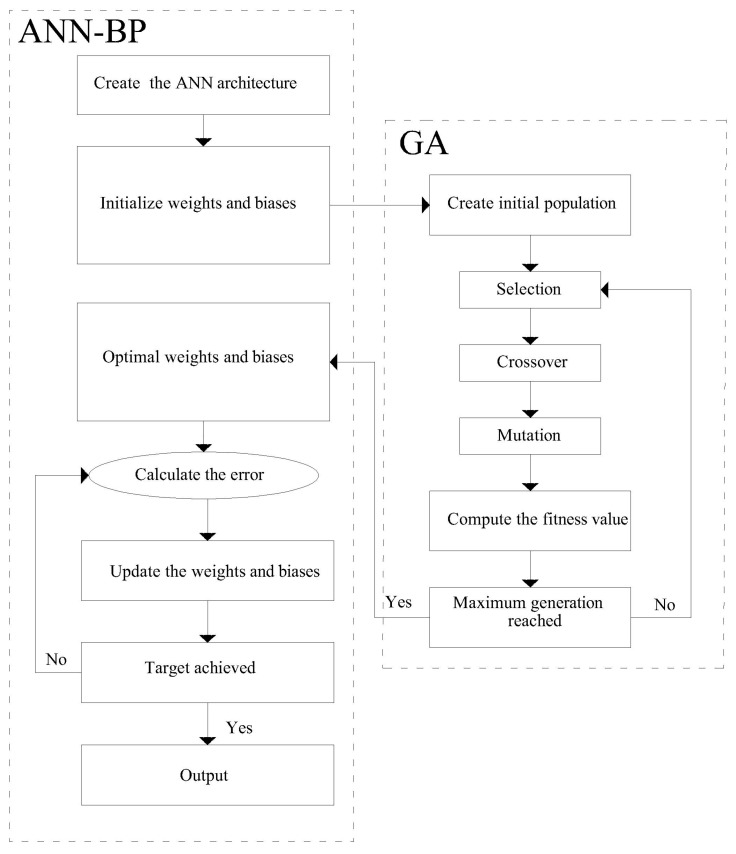
Flowchart of artificial neural network–back-propagation (ANN–BP) optimized by GA.

In the present paper, GA was applied to optimize the evolution of weights and biases as shown in Figure 3. Therefore, mutation and crossover only apply to the weights and biases to find the optimal values. After achieving optimal weights and biases, the model is trained using a BP algorithm.

There are several training paradigms for ANN. For instance, the training can be supervised or unsupervised, i.e., learning with a teacher or learning without a teacher [37,55,66]. In the case of supervised learning, the network is trained on certain provided data to a targeted output. Thus, the network can learn in an administered manner. Conversely, in the case of unsupervised learning, the network is guided to learn independently. Thus, it can recognize the analogy among the training pattern on its own.

In the majority of engineering applications of ANNs, the supervised training method based on feed-forward network along with a back-propagation algorithm was implemented [37,61,62,66]. According to Yeh [43], developing an ANN model capable of predicting the behavior of a material requires training the network on the targeted data obtained from experimental results of that material. In other words, it requires training the network in a supervised manner. Thus, in the present study, feed-forward neural networks (FFNs) along with back-propagation algorithm was implemented to train the GA–ANN model on predicting self-healing in concrete.

The GA–ANN model was trained using 70% of the total database, which was randomly selected to avoid any bias. It includes the influential parameters investigated in the selected studies. Moreover, 15% of the data (also selected randomly) was used for the validation of the model, while the remaining 15% was used for testing the generalization capability of the model. The validation data set is different from the model generalization test set. Validation is regarded as part of the training process. It is used to build the model and determine its parameters in order to avoid overfitting. The non-linear ANN model could get full accuracy on the training data set. This overfitting has been found to lead to very poor performance on the test data set. Hence, the independent validation data set is used for “cross-validation” to avoid such overfitting. Conversely, the test data set is only used to explore the performance of the trained model on new, unfamiliar data. In other words, the training data set is used for determining the ANN weights and biases to minimize the error function and maximize accuracy in each iteration. The cross-training data set is used to oversee the training process and improve the ANN generalization by minimizing overfitting. An overfitted ANN yields high accuracy on training data, yet fails to generalize from the training data, thus yielding poor performance on new, independent input data. The validation data set thus provides an unbiased estimate of the generalization error of the model.

The back-propagation Levenberg–Marqudt rule (LMA) was used to simplify and shorten the training time. It basically propagates back the calculated error at the output layer to the network based on the Jacobian matrix *J*. The iteration of such an algorithm can be written as follows:

(5) $$w_{j+1}=w_{j}-{\left[\;J^{T}J+µI\right]}^{-1}J^{T}e$$

where *w_j_* is a vector of current weights and biases; *µ* is a learning rate; *J* is the Jacobian matrix; *J^T^* is the transpose matrix of *J*; *I* is the identity matrix; and *e* is a vector of network errors.

### 4.4. Database Sources and Range of Input and Output Variables

As shown in Table 2, data were collected from various studies including Wiktor and Jonkers [27], Sisomphon et al. [11], Van Tittelboom et al. [17], Sahmaran et al. [54] and Özbay et al. [53]. The total database included 1462 data points. These reported studies indicated that crack self-healing of cementitious materials is controlled by factors including the binder content, w/c ratio, initial crack width and the healing time. For instance, Sahmaran et al. [54] and Özbay et al. [53] and Van Tittelboom et al. [17] investigated the influence of using alternative binder materials including supplementary cementitious materials on crack self-healing. Cracks were created in mortar cylinders by means of a crack width-controlled splitting test. It was found that cement partial replacement by blast furnace slag or fly ash improved the crack self-healing. In addition, decreasing the water-to-binder ratio improved the self-healing efficiency.

Sisomphon et al. [11] investigated the potential of promoting self-healing in cementitious materials through crystalline and expansive additives. They used synthetic cementitious materials made of reactive silica and crystalline catalysts as a crystalline additive and calcium sulfo-aluminate as an expansive material. It was found that, within 28 days, pre-cracked specimens were capable of self-healing their cracks with a width of up to 400 µm. Jonkers et al. [21] studied the effect of using a certain type of alkali-resistant spore-forming bacteria in a cementitious material. Lightweight aggregate impregnated with spore-forming bacteria related to the genus Bacillus was added into the cementitious material during the mixing time. Results showed that crack healing occurred for up to 460 µm-wide cracks in specimens incorporating the spore-forming bacteria compared to 180 µm-wide crack healing occurring in control specimens made with ordinary Portland cement.

Therefore, according to the reported studies above, 11 input parameters encompassing the factors controlling self-healing in cementitious materials were used as the input data to train the GA–ANN. As shown in Table 2, nine parameters representing the original mixture proportions in terms of Portland cement dosage (C), water-to-cement ratio (w/c), sand (S), fly ash (FA), ground granulated blast furnace slag (SL), expansive additive “cement-based material incorporating calcium sulfo-aluminate” (CSA), crystalline additive (CA), lightweight aggregate (LWA), and lightweight aggregate with bacteria spores (LWAB) were used as input data. The additional two parameters were the initial crack width (CWI) and the healing time (HT). The database of results of crack width change as a function with time due to self-healing obtained from these experimental studies were used as target output in the GA–ANN model. Thus, the corresponding final crack width (CWF) was used as a single output.

## 5. Performance of GA–ANN Model

**Figure 4:**
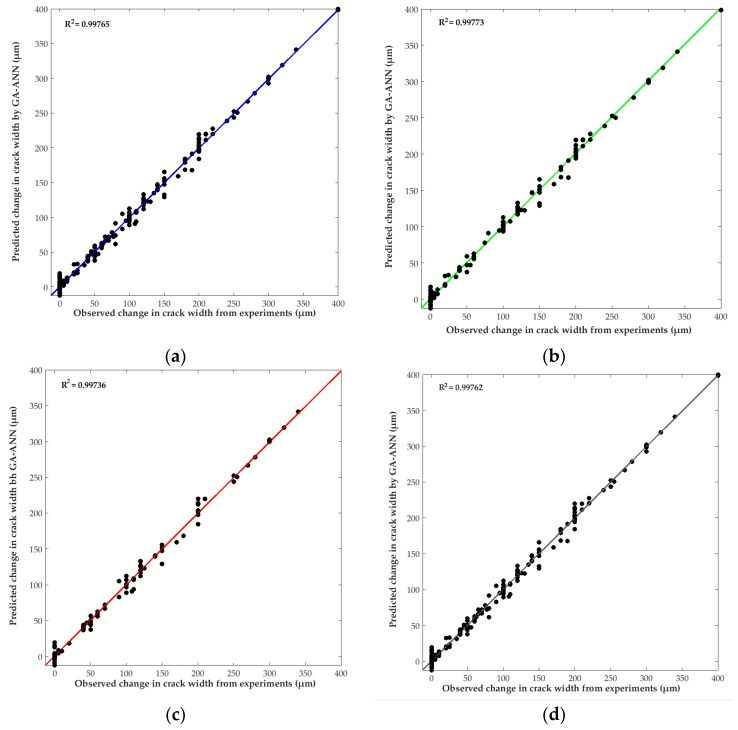
Regression plot of GA–ANN predicted change in crack width due to self-healing versus the corresponding experimentally observed change in crack width: (**a**) training; (**b**) validation; (**c**) test; and (**d**) complete data set.

Figure 4 exhibits the results of change in width of the self-healed cracks in concrete predicted by the proposed GA–ANN model versus the corresponding experimental measurements of crack self-healing reported in various studies. The performance of the GA–ANN model mainly depends on its ability to predict the experimental output data with reasonable accuracy. As shown in Figure 4, the GA–ANN model was able to accurately predict the self-healing of concrete relative to the actual experimental data. For instance, the coefficient of determination (*R*^2^) of model prediction versus experimental data for the training, validation, and test data sets are 0.99765, 0.99773, and 0.99736 respectively. Thus, it can be argued that the proposed GA–ANN model captured the relationships between the provided input and output data with adequate accuracy, which indicates excellent performance.

The reliability of the developed GA–ANN model for the complete data set was also evaluated via the root-mean-square (RMS) error, coefficient of determination (*R*^2^), and mean absolute percentage error (MAPE) between the model’s predictions and experimental results, according to Equations (6)–(8). The RMS, *R*^2^, and MAPE values were 10.19 µm, 0.99762, and 10.13%, respectively, which indicates adequate performance of the GA–ANN model.

(6) $$\mathrm{RMS}=\sqrt{\frac{1}{n}\overset{n}{\underset{i=1}{\sum }}{\left(t_{i}-o_{i}\right)}^{2}}$$

(7) $$R^{2}=1-\left(\frac{\sum_{i=1}^{n}{\left(t_{i}-o_{i}\right)}^{2}}{\sum_{i=1}^{n}{\left(t_{i}\right)}^{2}}\right)$$

(8) $$\mathrm{MAPE}=\frac{1}{n}\frac{\sum_{i=1}^{n}\left|t_{i}-o_{i}\right|}{\left|\sum_{i=1}^{n}t_{i}\right|}$$

where *t_i_* is the target output; *o_i_* is the predicted output; and *n* is the number of data point.

The generalization capacity of the GA–ANN model was also evaluated on randomly selected test data (15% of the original database), which was unfamiliar to the mode and not previously presented in the training process. The eleven input testing data were introduced to the GA–ANN model to predict the self-healing of concrete. As shown in Figure 4, the presented GA–ANN model was able to predict the self-healing of concrete relative to the actual experimental data. Furthermore, the model performance on the validation data was comparable to that of the training and testing data, again indicating adequate performance of the GA–ANN model in predicting the complex phenomenon of self-healing as a function of the multitude of interconnected governing variables.

**Figure 5:**
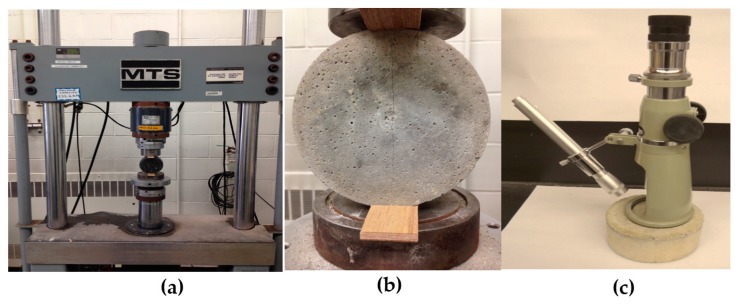
Crack development in mortar specimens tested for self-healing: (**a**) loading procedure; (**b**) crack development; and (**c**) crack width measurement using a microscope.

**Figure 6:**
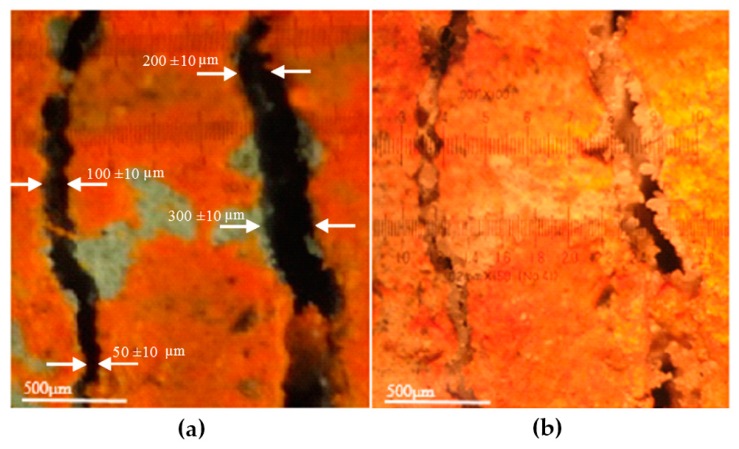
Crack healing process: (**a**) cracks before healing; (**b**) cracks after 42 days of healing.

**Figure 7:**
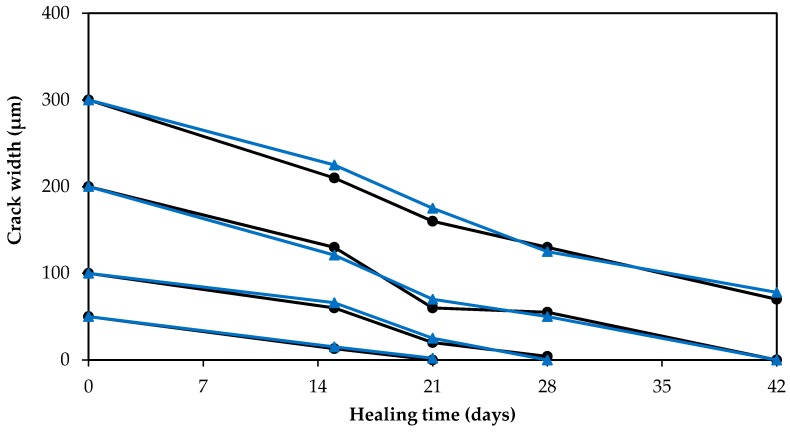
GA–ANN model predictions of crack self-healing (reduction in crack width) of cementitious materials versus corresponding experimentally measured results.

The ability of the GA–ANN model to predict the crack self-healing in cementitious materials was further validated using new experimental data obtained by the present authors in their lab. Cement mortar specimens were made with ordinary Portland cement partially replaced with 20% of fly ash. Water-to-cementitious materials ratio (w/c) of 0.35 and sand-to-cementitious materials ratio (s/c) of 2 by mass were used. The specimens were cast in plastic containers having a 4 cm diameter and 9 cm height. The specimens were reinforced with a galvanized steel mesh (6 mm × 6 mm with Ø = 1 mm). After 28 days of curing in a moist room at RH ≥ 95% and *T* = 21 ± 1 °C (68 °F), specimens were cracked by applying a tensile stress. Cracks with a width of 50 ± 10, 100 ± 10, 200 ± 10, 300 ± 10 µm, and 400 ± 10 µm were measured using an optical microscope and marked to evaluate the width change due to self-healing, as shown in Figure 5. All specimens were then submerged in water to allow the development of self-healing of cracks. The change in crack width was measured at 15, 21, 28 and 42 days wetting periods. Figure 6 illustrates the healing process of the cracked specimens. It was indicated that complete healing occurred in cracks with small width in comparison to cracks with larger width. The experimental results were compared with the GA–ANN model-predicted results. As shown in Figure 7, the GA–ANN model was capable of predicting the self-healing of cracks in the tested mortar specimens.

## 6. Conclusions

In this study, a hybrid genetic algorithm–artificial neural network (GA–ANN) model was developed to predict the self-healing of cracks in cement-based materials. From this study, the following conclusions can be drawn:

- The developed GA–ANN model represents a powerful computational tool with high efficiency providing an alternative solution for the modeling procedure of the highly complex self-healing phenomenon in cement-based materials.
- A genetic algorithm was effectively applied in the ANN model to determine the optimal weights and biases that govern the input–output relationship of the model.
- Training the GA–ANN multilayered feed-forward neural network with a back-propagation algorithm showed accurate prediction of the self-healing crack ability in cementitious materials, yielding predictions that were close to the actual experimental values.
- The proposed model was capable of providing accurate predictions for the self-healing ability of a cementitious material, which in return can be used to enhance the durability design of concrete, leading to more durable and sustainable structures.

## Figures and Tables

**Table 1:** Values of parameters used in GA–ANN modeling.

Parameter	GA–ANN
Number of input layer neurons	11
Number of first hidden layer neurons	14
Number of output layer neurons	1
MSE goal	13 × 10^−5^

**Table 2:** Database sources and range of input and output variables.

**Source**	**No. of Data Points**	
Wiktor and Jonkers [27]	640	
Sisomphon et al. [11]	594	
Sahmaran et al. [54]	36	
Van Tittelboom et al. [17]	182	
Özbay et al. [53]	10	
**Database Parameter**	**Maximum**	**Minimum **
Cement (mR %)	100	15
w/c (mR %)	60	25
Sand (mR %)	309	200
BFS (mR %)	220	0
FA (mR %)	220	0
Calcium sulfo-aluminate (mR %)	10	0
Crystalline additive (mR %)	4	0
LWA (mR %)	76	0
LWA with bacteria spores (mR %)	76	0
Initial crack width (µm)	400	40
Healing time (days)	150	0
Final crack width (µm) *	400	0

mR %: By % of mass ratio of cement. * Output variable.
